# Legal and Ethical Aspects of ‘Best Interests’ Decision-Making for Medical Treatment of Companion Animals in the UK

**DOI:** 10.3390/ani10061009

**Published:** 2020-06-09

**Authors:** Carol Gray, Peter Fordyce

**Affiliations:** 1 School of Law and Social Justice, University of Liverpool, Liverpool L69 7ZR, UK; 2 Department of Veterinary Medicine, University of Cambridge, Cambridge CB3 0ES, UK; psf23@cam.ac.uk

**Keywords:** best interests, veterinary treatment, companion animals

## Abstract

**Simple Summary:**

Making decisions about medical treatment for animal patients involves two key decision-makers, the animal owner and the veterinary surgeon. We aim to show that these decisions should and can be based on the ‘best interests’ of the animal, with both human decision-makers acting as advocates for the animal requiring treatment. We suggest that the role of the animal owner is similar to that of a parent in making decisions for a child, drawing on legal cases to demonstrate the limits of parental (and owner) decision-making. To provide a firmer basis for ‘best interests’ decision-making, we adapt the factors included in the United Nations Convention on the Rights of the Child and demonstrate how these could be used with a typical clinical situation. Finally, we analyse the decisions from an ethical point of view.

**Abstract:**

Medical decisions for young children are made by those with parental responsibility, with legal involvement only if the decision is potentially detrimental to the child’s welfare. While legally classified as property, some argue that animals are in a similar position to children; treatment decisions are made by their owners, posing a legal challenge only if the proposed treatment has the potential to cause harm or unnecessary suffering, as defined by animal protection legislation. This paper formulates the approach to a ‘best interests’ calculation, utilising the factors included in the United Nations Convention on the Rights of the Child and relying on exchange of information between the human parties involved. Although this form of decision-making must primarily protect the animal from unnecessary suffering, it recognises that the information provided by the owner is critical in articulating the animal’s non-medical interests, and hence in formulating what is in the animal’s best overall welfare interests. While statute law does not mandate consideration of ‘best interests’ for animals, this approach might reasonably be expected as a professional imperative for veterinary surgeons. Importantly, this version of a ‘best interests’ calculation can be incorporated into existing ethical frameworks for medical decision-making and the humane treatment of animals.

## 1. Introduction

According to Coggon, for medical treatment to be lawful, it requires that treatment is deemed to be in the patient’s best interests (in the opinion of the medical professionals involved); that it aligns with the patient’s values (demonstrated, for example, by the patient giving informed consent); and that the necessary resources (including finances) are available to provide the treatment [1]. Consequently, for adult patients with capacity to consent, the legality of treatment is unequivocal. However, for patients without capacity, the picture is somewhat obscured by attempts to make decisions in their ‘best interests,’ when the latter term is regarded as being vague, subjective, and too difficult to determine [2,3,4].

For adult patients without capacity, whether the loss of capacity is permanent or temporary, defining a ‘best interests’ basis for treatment decisions involves, at some stage, incorporating their values and wishes in the calculations, as demonstrated in several high-profile cases in the United Kingdom (UK), for example, in ‘Aintree University Hospitals NHS Foundation Trust v James’ [5] and in ‘Briggs v Briggs’ [6], where the patients’ previously expressed wishes were pivotal to the eventual ‘best interests’ decisions. For those adult patients lacking capacity, for example, due to severe learning disabilities, the UK Mental Capacity Act 2005 requires that they should be supported to make decisions wherever possible; they may have capacity for some decisions and not for others. Thus, adult patients without capacity may not be suitable as a comparison for animal patients. In view of the problem with finding out what animals want, particularly if relying on human interpretation of their values and preferences [7], it is more usual to compare the situation of the animal patient with that of the infant child, whose values and preferences are not yet known [8,9]. This comparison, although enticingly straightforward, must incorporate an acknowledgement that, eventually, animal emotions and desires will be more accurately interpreted by humans, perhaps allowing determination of the individual animal’s preferences with regards to the life circumstances in which it finds itself. Such interpretations will also depend on the developing knowledge from animal welfare science that underpins the concept of ‘Critical Anthropomorphism’ [10]. Importantly, the difference in legal status between children and animals must also be appreciated, notably the animal’s position as property.

For both paediatric and veterinary patients, the decision-maker is also the carer. By limiting the comparison to companion animals, we focus on animals that are frequently regarded as family members [11], with decisions made for them accordingly. Unlike decision-making for farm animals or laboratory animals, where interventions are mainly for the benefit of (human) individuals or society, the authors argue that, ideally, decision-making in companion animals should be made on the basis of the ‘best interests’ of the individual animal [12]. For children, decisions are made by an adult with parental responsibility. For animals, decisions are made by the person legally regarded as the owner, or the owner’s agent. Although many scholars are critical of the use of the term ‘ownership’ with regards to animals, we will use this term to denote the person with primary caring responsibility for an animal.

We undertook the comparison by researching UK case law regarding medical decision-making for children, focusing on judgments containing key references to the best interests of the child. We scrutinised UK child and animal welfare legislation and utilised the United Nations (UN) Convention on the Rights of the Child as a potential source of a ‘best interests’ calculation.

Direct comparisons between paediatric and veterinary decision-making require specific assumptions; first, that the owner regards the animal as having intrinsic value, and second, that there is an ultimate ethical acceptance of the subservience of the owner’s potential ‘selfish interests’ in the relationship with the animal to the best interests of that animal. Because of the different legislation and funding arrangements for treatment that protect children and animals from harm, this ‘selfish interest’ concept may include resolution of the dilemmas surrounding the ability or desire of the owner to fund any potential treatment. Ultimately the outcome of what is in the animal’s best interests is likely to depend on the dialogue between the veterinary surgeon, who has the technical and legal knowledge relating to the animal’s health and welfare, and the owner/carer, who is able to inform the discussion regarding aspects of the animal’s wider welfare, its individual circumstances and temperament.

Bridgeman observes that decision-making for children involves two dependencies: first, the parent’s dependence on the healthcare professional for information and professional advice and second, the child’s dependence on the parent for care and responsible decision-making [13]. While recognising that similar dependencies may apply to decision-making in veterinary medicine in the UK, given the ethical imperative explicit in the oath sworn by Members of the Royal College of Veterinary Surgeons (MRCVS) “that, ABOVE ALL, my constant endeavour will be to ensure the health and welfare of the animals committed to my care” [14], the authors argue that the role of the two human parties to the veterinary treatment decision should be for both to advocate for the animal patient, enabling a decision that is in the animal’s best interests in its individual medical situation and practical circumstances. Nevertheless, any theoretical advocacy must be considered in the practical context of the legal protection constraining the level of harm the animal has to endure.

Both paediatric and veterinary decision-making are constrained by baseline protection for the patient. For children in the UK, protection against cruelty and unnecessary suffering is still provided under the auspices of the Children and Young Persons Act 1933, although in other areas, much of this legislation has been replaced by the provisions of the Children Acts 1989 and 2004. Protection for animals in the UK is provided by the Animal Welfare Act (AWA) 2006 (as devolved), which replaced the Protection of Animals Act 1911. Both sets of legislation prohibit anyone (not just the parent or owner) from treating the child or animal cruelly. For medical scenarios, there are two likely applications of these welfare protection laws:

(1) To ensure that the patient is given any necessary treatment, i.e., to rule out the option of no treatment (while acknowledging that refusal of treatment would be accepted as a decision for adult patients with capacity to make such a decision).
(2) To prevent the carrying out of any treatment that may cause harm or unnecessary suffering (for animals, this would include prohibited mutilations, and experimental medical treatments not considered to be ‘Recognised Veterinary Practice’ by the RCVS [15]).

In the case of the AWA 2006, the requirement to provide treatment is encompassed in Section 9, where a person commits an offence “if he does not take such steps as are reasonable in all the circumstances to ensure that the needs of an animal for which he is responsible are met to the extent required by good practice”, one such need being: “its need to be protected from pain, suffering, injury and disease” (albeit with caveats in SubSection 3).

The legal liabilities incumbent on both owner and veterinary surgeon under Sections 3, 4 and 9 of the AWA act as constraints to decision-making, not least the fact that killing or euthanasia of the patient are legal options open to the animal’s owner under Section 9, and that due to liabilities in Section 3, Coggon’s issue relating to resource provision may have a significant impact on the practical outcome of where ‘best interests’ lie in a specific situation.

An egregious breach of Section 9 may lead to a prosecution under Section 4 for ‘unnecessary suffering.’ While what constitutes ‘suffering’ under the Act is vaguely defined [16,17], there is guidance in Section 4 (3) on what a court might consider ‘unnecessary.’ Such considerations include whether the suffering was for the longer-term benefit of the animal. As pointed out by Grimm and others, while any clinical treatment can cause harm, if it can be justified in the animal’s health-related interests, it can be justified under the Act [18]. However, other caveats also apply to this principle; any suffering caused by the treatment must reasonably be avoided, or reduced, and be proportionate to the purpose of the conduct.

Importantly, animal welfare protection legislation in the UK, despite having recently progressed from a resolutely negative (what an owner must not do to an animal) to a more positive (what an owner must provide for an animal) stance, still fails to promote the best interests of the animal (as exemplified by Subsections 3 and 4 of Section 9, which allow for lawful use and humane destruction). As Robertson notes, animal protection legislation focuses on minimum standards rather than mandating ‘best practice’ [19]. Therefore, consideration of decision-making that prioritises the best interests of the animal under veterinary care requires a substantial input from the field of veterinary ethics, as outlined in the development of the veterinary ethical tool by Grimm and others [18]. While the latter authors centre ‘best interests’ as their moral foundation for decision making in companion animal practice, based on restoration of the animal’s health while aiming for a positive balance of quality of life, factors other than Bridgeman’s first dependency (the parent/carer’s reliance on the healthcare professional for information and professional advice) are clearly key to establishing what an animal’s ‘best interests’ are in its particular circumstances.

If Grimm and others’ second imperative (to respect the animal’s quality of life experience) is to be upheld, information regarding the animal’s temperament and character, the (owner-provided) physical environment, and owner-related factors such as knowledge, ability, and financial and time resources may all impact on the animal’s ‘quality of life’ experience.

In adding the preservation of the human-animal relationship to this list, Schnobel proposes that the primary role of the veterinary professional may be to provide the owner with the information about proposed treatment(s), with a view to maintaining the ideal human-animal relationship, enabling the animal to “participate within a companion relationship between owner and animal where both derive a significant benefit” [20]. In common with many other lists, the preceding examples maintain a degree of flexibility and individual interpretation that can frustrate attempts to base decisions on ‘best interests.’ Therefore, in seeking to identify specific factors that may constitute the calculation of the ‘best interests’ of an animal patient, we now turn to examine the lessons that can be learned from the field of children’s rights and best interests.

## 2. Factors Included in a ‘Best Interests’ Calculation—the Child and Animal Comparison

For children, the best interests test originated as a legal requirement in child custody cases at common law and was later applied to paediatric healthcare [21]. A major problem with an objective ‘best interests’ standard is the difficulty of defining ‘best interests, which, according to Baines, may be ontological (there may be no such thing as objective best interests), or epistemological (best interests may exist, but there is no way of discovering what they are) [22].

Section 1.3 of the UK Children Act proposes a list of factors which courts should take into account in determining the best interests of the child, including the risk of any harm, and assessing the emotional as well as physical needs of the child. However, it seems that in many UK cases involving children, the avoidance of harm and the ‘reasonableness’ of the decision is not enough. Some early cases took the approach that a procedure may be performed provided it would not harm the child [23], but this has been firmly rejected, first by the Court of Appeal in the Charlie Gard case, where the child’s parents, seeking to take him overseas for treatment with an unproven therapy, appealed on the grounds that the judge had erred in preventing such treatment when there was no risk of the treatment causing significant harm to the child. Dismissing the appeal, McFarlane LJ reinforced the ‘best interests’ standard and refused to create a “subset of cases based upon establishing significant harm” [24]. This ruling was endorsed by the Supreme Court in the Alfie Evans case [25], where the court unanimously dismissed an argument made by the parents of a terminally ill child that the appropriate test for their request to take their child to Italy for treatment should be that such an intervention would not cause significant harm, even if it were not in the child’s best interests.

Thus, the ‘gold standard’ now applies a positive version of best interests (a decision should positively promote the interests of the child) rather than a negative version where the focus is on the avoidance of harm. The ‘gold standard’ should also extend to more than just medical interests, as illustrated in the ‘Re T’ case, where a child’s best medical interests would have been served by undergoing a proposed liver transplant. However, the Court of Appeal overturned the decision in the lower courts, which had found for the medical professionals based on the ‘unreasonableness’ of the mother’s refusal to consent. In her judgment, Butler-Sloss LJ stated that “to prolong life … (…) … is not the sole objective of the court and to require it at the expense of other considerations may not be in a child’s best interests” [26]. Therefore, a ‘best interests’ decision for children cannot be based solely on the preservation of life, meaning that extension of life at any cost to the child’s welfare is of itself not a justification for medical intervention, nor can it be based solely on the avoidance of harm.

Turning to the animal patient, the authors are concerned that many veterinary decisions are based on precisely these two parameters, by considering ‘extension of life’ and ‘avoidance of harm’ to the exclusion of other factors that should inform a “best interests” decision. Moreover, in animals, there is considerable debate over the place of death in these calculations. Authors such as Webster [27] and Broom [28] do not consider death to be a harm to an animal’s welfare, as once the animal’s brain has ceased to function, it clearly will have no awareness of any feelings that could be described as aversive mental states, i.e., suffering. However, others would argue that suffering is not the only harm that can be caused to an animal; death is also potentially a harm, by depriving the animal of the possibility of experiencing positive mental states (pleasure) through continuing to live [29,30].

Harms can involve suffering or deprivation. Rollin regards pain as the worst harm that can be inflicted on an animal, due to the perceived inability of animals to anticipate the end of their suffering [31]. Thus, although death can be regarded as the ultimate deprivation, it may not be the worst harm [32]. Expanding on this argument, if “the presence of a life” has positive value to the animal then death is a harm, but conversely if that life has negative value then death is a benefit [29]. End-of-life decision-making in veterinary medicine prioritises the prevention of suffering, requiring that in cases of poor welfare, the decision is made for euthanasia [33]. In agreeing with the priority given to avoidance of pain in these situations, Linzey proposes that, faced with a choice between the duty to preserve life and the duty to prevent suffering, the second duty should take precedence [34]. Therefore, decision-making in animals tends to prioritise the avoidance of harm. Although it has been rejected as the basis for decision-making for babies with life-limiting conditions, where the intention to preserve life must take priority, input from a ‘harm avoidance’ perspective helps to define the limits to ‘best interests’ decision-making for both children and animals.

Grimm and others’ ethical framework for decision-making for veterinary patients incorporates the idea that short-term harm for long-term best interests can be justified utilising a similar approach to that used to justify research on animals [18]. In this framework, the veterinary surgeon is placed at the centre of the process, first deciding whether the proposed treatment is in the best interests of the animal. This decision is made using criteria such as improving health, improving quality of life, minimising harm and performing a harm-benefit analysis. Only after evaluating these parameters is there a discussion of client-associated (secondary) factors such as effect on client quality of life and the owner-animal relationship. Although this tool could prove useful when considering cutting-edge or novel therapies, we consider that it neglects the contribution of the owner to the ‘best interests’ discussion for more conventional treatments. We therefore propose that basing the decision on calculations of ‘best interests’ for children may lead to a more universal approach for veterinary treatments. We now attempt to utilise an existing framework, developed for children, to define the contents of a ‘best interests’ discussion for an animal patient.

## 3. Using the UN Convention on the Rights of the Child as a Framework for Calculating Best Interests for Animals

While positing an approach that leaves the determination of ‘best interests’ open to interpretation, and lauding the flexibility of such an approach, the UN Convention on the Rights of the Child (CRC) proposes several factors for inclusion in any ‘best interests’ calculation. In its accompanying document, General Comment 14 [35], the CRC includes a (non-exhaustive) list of factors to be considered when compiling a ‘best interests’ calculation, some of which may be suitable for inclusion in a similar list for animals. These are included in Table 1.

Although, at first glance, “the child’s views” and “the child’s identity” seem uniquely applicable to children rather than animals, they are in fact worthy of closer examination. In light of expanding knowledge of animal preferences, there may be a place for inclusion of the animal’s views in the list. In suggesting that the owner may be best placed to report on the animal’s personality and preferences, we are highlighting that, in a similar role to those with parental responsibility, animal owners have caring responsibilities that give them unique insight to the animal’s world. Nevertheless, assigning the role of interpreter of animal preferences to the animal owner may add to the ‘caregiver burden’ felt by many owners [36]. Additionally, a number of caveats need to be applied to the owner’s perception of the state of their animal’s welfare [37,38]. These caveats include assessing whether the owner has failed to observe and report physical signs in the pet that the veterinary surgeon would perceive as indicators of poor welfare, for example, obesity [39], or whether having noticed these signs, the owner does not consider them to constitute a welfare problem, for example, breathing difficulties in brachycephalic dogs [40]. Hence, while the owner’s intent towards their animal’s welfare may be beneficent, their interpretation of the state of its welfare should be caveated with a professional assessment of welfare, followed by an appropriate discussion with the owner.

Nevertheless, the authors would argue that consideration of the owner’s interpretation of what is in the animal’s best interests should stand as a ‘prima facie’ principle in the decision-making process, and only then should each owner-animal dyad be scrutinised on an individual basis.

Subsequent factors appear more straightforward to relate to the veterinary context. “Preservation of the family environment and maintaining relations” foregrounds the importance of the relationship between the patient and their carer. As Bridgeman [13] asserts, most parents seek to ‘do their best’ for their children, therefore, in most cases, parents are the most appropriate decision-makers, as was observed by Baker J in ‘Re Ashya King (a child)’:

“… the parents are the best people to make decisions about a child and the State … (…) … has no business interfering with the exercise of parental responsibility unless the child is suffering or is likely to suffer significant harm as a result of the care given to the child not being what it would be reasonable to expect a parent to give.”

[41]

In applying the same test to animal owners, we propose that they are the best people to make decisions about their charges, unless there is clear evidence that the animal is suffering or is likely to suffer harm. Of course, there are some owners who do not prioritise the animal’s interests, however we maintain that such owners are in the minority [42,43]. Importantly, some owners may be unable to prioritise the animal’s interests due to financial constraints that restrict the choices available to them.

The fundamental requirement to preserve the “care, protection and safety” of the child or animal patient engages the main principles of welfare legislation, with more positive responsibilities to provide for the patient’s basic welfare needs.

It could be stated that a “situation of vulnerability” applies to all animal patients, although its application in the CRC is limited to children with disabilities, those who are refugees, belong to a minority group or are victims of abuse. Certainly, rescued and abused animals would fit this category, and the suggestion that their interests should be specifically analysed seems appropriate. This situation would prioritise ‘protection from harm’ as a key area for discussion regarding such patients.

Two basic rights, to health and education, complete the CRC’s list of factors that must be considered for a ‘best interests’ calculation in children. While education seems less relevant for animals (unless translated as training, but that may be stretching the comparison), the right to health could be given prominence, although such an assignment may unfairly prioritise health-based interests above all others. Additionally, switching to a rights-based narrative may invoke protest. Conversely, in light of the perceived problems with assigning rights to animals, a ‘best interests’ approach may achieve what a rights-based agenda could not. Even children’s rights are not universally accepted, and as Kilkelly observes, “without rights language, the child’s best interests appears as a conciliatory gesture with its softer language reaching out to those to whom rights are not acceptable, whatever the basis of that position” [44].

Assigning a right to health to the animal through a ‘best interests’ calculation could perhaps move the argument further along the road for animal rights. Indeed, the veterinary profession’s “self-imposed ethical ideal of advocating and defending its patients’ interests in health” [12] already gives the animal patient status as a subject.

## 4. A Practical Approach to a ‘Best Interests’ Calculation for Animals

Any ‘best interests’ calculation, whether for children or animals, necessarily involves two key aspects. The first is the medical knowledge of the healthcare professional, translated into accessible information for the caregiver. The second, which invokes the relational responsibility to which Bridgeman [13] alludes, is the unique personality of the patient, interpreted by the caregiver as the patient’s preferences and values. In the authors’ opinion, these two (human) agents should have equal input to the discussion surrounding ‘best interests,’ which could be achieved by incorporating the ideals of collaborative decision-making [45]. Such collaboration between veterinary healthcare professionals and animal owners requires discussion of the financial burdens of treatment, which may impact on the choices available. Where an owner is unable to afford the treatment required, the private enterprise veterinary healthcare market dictates that the options available may include severance of the ties between owner and animal.

To illustrate the stark choices involved, we now consider the hypothetical case of a nine-month-old neutered female cat who has been involved in a road traffic accident, sustaining several fractures that will require surgical repair. The patient has been made comfortable, while the owner and veterinary surgeon discuss the options. The owner has stated that he cannot afford the proposed surgery and is not eligible for referral to a veterinary charity for treatment.

The options under consideration are:

(1) The practice will perform the surgery at a vastly reduced rate, or offer a payment plan that fits with the owner’s financial situation.
(2) The owner will relinquish ownership of the animal to the practice, with an agreement that the cat will be rehomed once the surgery has been successfully carried out.
(3) The owner will agree to euthanasia of the cat.

These options can be evaluated in more detail with a principal focus on the ‘best interests’ of the cat, relating these to similar provisions in the CRC.

We propose that Option 3 may prevent harm by ending suffering, but it would also deprive the cat of the ability to experience a ‘life worth living’ for a member of her species. We would argue that euthanasia in these circumstances cannot be said to be in the cat’s best interests, as it neglects her “care, protection and safety,” her “right to health,” and, from previous ethical discussion, her ability to experience pleasure in the future. Additionally, this option would sever the unique owner-companion animal relationship.

Option 2 would also entail the severance of the human-animal relationship. In the hypothetical scenario, the owner rescued the cat as a six-week-old kitten, and they have a bonded relationship that seems to give the cat (and owner) pleasure. Moreover, the cat enjoys exploring her owner’s garden and the local neighbourhood, where there are few other cats. Therefore, we propose that a “best interests”-based decision would need to “preserve the family environment and maintain relations” and would not contemplate removal of the cat from her current living arrangements.

Thus, we consider Option 1 as unique in preserving the current relationship, which seems to be of value to the cat, thus respecting her “views and identity.” Following a period of recovery from surgery, the cat will return to her home territory, which is the ideal outcome from the perspective of her ‘best interests’.

Note that in selecting the option that prioritises the cat’s best interests, we have ignored the interests of the veterinary practice as a business entity, and have assumed that the veterinary staff involved will also prioritise the interests of the cat over personal financial interests, such as practice profit or receiving bonus payments on turnover. However, both Options 1 and 2 involve financial loss to the practice on a similar scale, so perhaps the real choice is between performing the surgery, regardless of ownership, and euthanasia. The discussion of ‘best interests’ in the context of financing veterinary treatment illustrates how ethical dilemmas resulting from multiple competing interests require consideration and resolution.

Such dilemmas can be viewed through the perspective of differing normative ethical systems such as utilitarianism (looking at what would be the greatest good for the greatest number of moral agents and patients involved); deontology (examining the duties owed by the parties to each other); virtue ethics (assessing the actions of the moral agents involved in relation to a vocabulary of virtues and vices such as ‘compassionate’ or ‘cruel’); rights discourse (examining what legal and moral rights or entitlements the parties have, including the right of an individual involved to not have their interests overridden by utilitarian claims); and relational and care ethics (arguably a branch of virtue ethics, which places the nature of the interpersonal relationships between the various parties at the centre of the moral argument). However, no one normative ethical perspective is likely to provide a calculus resulting in a morally acceptable conclusion for all parties; for example, utilitarian perspectives may violate deontological imperatives for individuals not to be treated ‘as a means to an end’, while ‘rights discourse’ may infringe on concepts from virtue ethics such as fairness, when considering distribution of resources such as financing the surgery discussed above.

Perhaps unsurprisingly, two of the most relevant contemporary ethical frameworks are associated with human patient care, and the use of animals as a food source by humans. These frameworks incorporate multiple elements from normative ethics, to allow not only consideration of the interests of the moral agents and patients involved, but also the consideration of these interests from the perspective of different ethical approaches.

While not universally accepted, the framework of ‘Principlism’ is a dominant ethical approach used in human medicine to highlight, and help resolve, ethical dilemmas in medical decision making [46]. Principlism asks the clinician to consider four ‘prima facie’ principles (principles that stand, until found to be in conflict with another principle) within the wider context of the patient’s situation (scope). The principle of ‘patient autonomy’ provides a deontological perspective, ‘beneficence and non-maleficence’ a utilitarian perspective, while ‘justice’ incorporates concepts from virtue ethics, consideration of legal and moral justice, fairness in relation to resource distribution, and concepts from ‘care ethics.’ Scope provides a perspective of the potential effect of the decisions made on all parties likely to be affected and might be considered to incorporate aspects of communitarian ethics [47].

The second framework, Mepham’s Matrix, was developed to consider the competing interests of those involved in production of animals for food, including the animals themselves [48]. This matrix provides a format to consider the impact of different decisions on various stakeholders, thus approximating scope, and examines three areas of ethical concern: the impact on the stakeholders’ wellbeing, including health and welfare (utilitarian perspective); autonomy, including freedom and choice (deontological perspective); and whether the decision would be seen as just and fair to them (virtue ethics perspective). As has been discussed elsewhere by both authors [49,50], the issue of using ‘autonomy’ as a prima facie principle in the context of animals is problematic, not least given the anthropocentric weight that tips the scales in favour of human benefit when discussing the moral value and utility of animals, a point acknowledged by the Food Ethics Council. Such inconsistency between the moral value of humans and animals is reflected in statute legislation, as previously discussed. However, replacing the principle of ‘autonomy’ with ‘best interests’ in the Principlism paradigm avoids many of the issues with interpretation of autonomy for animals. Specifically, it avoids the debate over whose autonomy should be respected; the owner’s autonomy, which is already constrained by animal welfare legislation and financial limitations, or the animal patient’s autonomy, which is difficult to interpret with current knowledge about animal cognition and behaviour. At the same time, it highlights the importance of the moral standing of the veterinary patient that might be expected in the light of the veterinary surgeon’s professional ethical obligations.

Grimm and others’ veterinary ethical tool [18] adds much to the debate in terms of examining beneficence and non-maleficence with regard to treatment options, in particular the avoidance of ‘futile’ treatment. However, the authors would suggest that while much of the focus of their approach is on the animal’s health, which is a major contributor, welfare encompasses much more than just health. The concept of ‘best interests’ enables this wider perspective to be considered. Such analysis requires considerable input from the animal’s primary caregiver, both in relation to issues such as the animal’s nature and temperament, its likes and dislikes, pleasures and fears, as well as the nature of the resources the caregiver is able (or willing) to bring to bear to achieve those best interests.

In considering the clinical scenario described above for the cat involved in the road accident, substituting the concept of ‘best interests’ for ‘autonomy’ as a prima facie principle in the principlism paradigm makes this central to any debate in relation to the other principles, should these clash at a practical level. Such an approach upholds the veterinary surgeon’s professional responsibilities to the oath sworn on admission to the profession. Additionally, it allows the wider welfare issues surrounding the animal’s wellbeing to be discussed outside the purely technical issues of the veterinary surgeon’s area of health and medicine.

Issues such as the good or harm a treatment (or lack of treatment) may cause, and particularly discussions about futile treatment can be raised within the ‘beneficence/non-maleficence’ principles. Similarly, issues relating to legal and moral rights and duties, as well as fairness with regard to issues such as funding treatment and breaking of relationships would fall under the principle of justice.

Clearly, any ethical framework is unlikely to function as an algorithm to provide a ‘morally correct output’ that is accepted by all parties affected by the decision; a point highlighted by the Food Ethics Council. It should, however, highlight the interests of all involved, including the responsibilities of the moral agents to the law, professional ethical obligations, and ethical duties as an owner. Where moral dilemmas arise due to a clash of prima facie principles, rational discussion can then take place about the way forward. The ‘best interests’ principle ensures that the wider wellbeing of the veterinary patient is at the centre of the discussion.

In the case of the cat discussed in the clinical scenario above, Option 1 (performing the surgery at a reduced rate or offering a suitable payment plan) appears to be in the ‘best interests’ of the animal. While Option 2 (relinquishing ownership) may meet the test for beneficence and non-maleficence in the narrower context of the animal’s health, it clashes with the principle of ‘best interests’ for the animal for the reasons discussed above and would likely have a negative psychological impact on the owner. Similarly, while Option 3 (euthanasia) may address the welfare issue of the cat’s current suffering, as once dead, she can no longer suffer, it deprives the cat of the potential of a favourable balance of pleasure over suffering during her life if treated successfully. When the decision is considered in terms of ‘scope’, it would also have a negative psychological impact on the humans involved.

The ethical dilemma arising from this scenario is the tension between what is in the patient’s ‘best interests’ and the principle of ‘justice’ with regard to paying for the treatment. Such dilemmas may well depend on the individual circumstances of the case, and disagreement may arise over what is considered ‘ethically acceptable’. The Farm Animal Welfare Council (FAWC) provides some guidance on ethical disagreements, suggesting that confidence in the validity and worth of the conclusion depends on three criteria:

(1) The arguments that lead to the particular conclusion are convincingly supported by facts, scientific deductions, and reason;
(2) The arguments are conducted within a well-established ethical framework;
(3) A considerable degree of consensus exists, arising from a process of genuine discussion and debate, about the validity of the conclusions [51].

In relation to FAWC’s first point, the concept of ‘best interests’ allows facts relating to the individual animal and its circumstances to be incorporated into the decision-making process by the owner and discussed in conjunction with the medical knowledge of the clinician. Inclusion of such information is therefore more likely to produce an ethically valid outcome for the parties involved.

As discussed, evaluating exactly what is in an animal’s wider best interests (not just its immediate health interests) must involve significant input from the owner, all be it caveated by the individual circumstances of the owner’s knowledge and understanding of the wider concept of welfare, and assessment of its translation to the individual pet at the time. We propose that the framework used in the UN CRC provides useful insights into how questions relating to the ‘best interests’ of a veterinary patient might be addressed in the conversation between the veterinary professional and the patient’s owner.

## 5. Conclusions

The use of a ‘best interests’ calculation has been prioritised in decision-making about the medical treatment of young children. In considering whether a similar calculation would be appropriate for animals, we have questioned the basis for such a calculation, drawing on the parameters advocated by the Convention on the Rights of the Child. The multiple difficulties inherent in such an approach, not least the disparity in legal status between children and animals, make such a comparison problematic. Moreover, the calculation of best interests for animal patients is heavily reliant on the human interpretation of animal preferences and values, and the willingness and ability of the human carer to pay for any required treatment.

In producing a draft set of criteria for consideration when attempting to calculate the ‘best interests’ of an animal patient, we have deliberately left some criteria open to interpretation, resisting the temptation to produce a ‘step-by-step’ guide. Such an approach fits with the intention of the UN CRC, which, in drawing up a ‘non-exhaustive’ and ‘non-hierarchical’ list of factors to be taken into account, intended the guidelines to be flexible. A key factor to the success of this approach is the importance of good communication skills. The veterinary surgeon must be able to communicate appropriately with the client to facilitate a ‘best interests’ discussion.

Importantly, with the involvement of at least two human advocates, a ‘best interests’ approach to veterinary decision-making gives the animal patient the priority that has perhaps been absent from veterinary medicine until relatively recently. The acknowledgement that we are discussing ‘best interests’ as a basis for veterinary decision-making, not to mention comparing it to medical decision-making for young children, requires elevation of the status of the animal patient to more than just ‘property’ or legal ’object’. Furthermore, as our knowledge of animal cognition and behaviour increases, the possibility of incorporation of the animal’s views in such calculations may become a reality.

The underpinning of ‘best interests’ discussions with previously posited ethical frameworks lends more weight to their use, with the term ‘best interests’ used in place of ‘autonomy’ when invoking human medical frameworks. The animal patient is thus given a position of moral relevance in veterinary science that fits with emerging views on how we decide on appropriate and ethical veterinary treatment.

Finally, through these endeavours, we propose that the animal patient, although currently lacking the status of legal subject, deserves to be considered a moral subject whose interests are worthy of primary consideration by the humans involved in making decisions on her behalf.

## Figures and Tables

**Table 1:** The components suggested for a “best interests’ calculation for children with interpretation for companion animal patients.

UN CRC Elements for ‘Best Interests’ Calculation	Possible Interpretation for Animal Patients
(a) the child’s views	human carer could interpret animal’s preferences
(b) the child’s identity	not applicable
(c) preservation of the family environment and maintaining relations	maintaining human-animal relationships and those with conspecifics
(d) care, protection and safety of the child	ensuring well-being and protection from abuse
(e) situation of vulnerability	special concerns for abused and stray animals
(f) the child’s right to health	advantages of treatment weighed against risks and side effects
(g) the child’s right to education	not applicable

## References

[1] Coggon J. (2016). Mental capacity law, autonomy, and best interests: An argument for conceptual and practical clarity in the Court of Protection. Med. Law Rev..

[2] Bester J.C. (2019). The best interest standard and children: Clarifying a concept and responding to its critics. J. Med. Ethics.

[3] Lim C.-M., Dunn M.C., Chin J.J. (2016). Clarifying the best interests standard: The elaborative and enumerative strategies in public policy-making. J. Med. Ethics.

[4] Wade D.T., Kitzinger C. (2019). Making healthcare decisions in a person’s best interests when theylack capacity: Clinical guidance based on a review of evidence. Clin. Rehabil..

[5] Aintree University Hospitals NHS Foundation Trust v James UKSC 67. https://www.supremecourt.uk/cases/docs/uksc-2013-0134-judgment.pdf.

[6] Briggs vs. Briggs EWCOP 53. https://www.bailii.org/ew/cases/EWCOP/2016/53.html.

[7] Franks B. (2019). What do animals want?. Anim. Welf..

[8] Rollin B. (2002). The use and abuse of Aesculapian authority in veterinary medicine. J. Am. Vet. Med. Assoc. (JAVMA).

[9] Ashall V., Millar K.M., Hobson-West P. (2018). Informed consent in veterinary medicine: Ethical implications for the profession and the animal “patient”. Food Ethics.

[10] Morton D., Burghardt G., Smith A. (1990). Critical anthropomorphism, animal suffering, and the ecological perspective. Hastings Cent. Rep..

[11] Charles N. (2016). Post-human families? Dog-human relations in the domestic sphere. Sociol. Res. Online.

[12] Weich K., Grimm H. (2018). Meeting the patient’s interest in veterinary clinics. Ethical Dimensions of the 21st Century animal patient. Food Ethics.

[13] Bridgeman J. (2007). Parental Responsibility, Young Children and Healthcare Law.

[14] Royal College of Veterinary Surgeons Declaration on Admission to the Profession. https://www.rcvs.org.uk/setting-standards/advice-and-guidance/code-of-professional-conduct-for-veterinary-surgeons/#declaration.

[15] Royal College of Veterinary Surgeons Code of Professional Conduct: Supporting Guidance 25: Recognised Veterinary Practice. https://www.rcvs.org.uk/setting-standards/advice-and-guidance/code-of-professional-conduct-for-veterinary-surgeons/supporting-guidance/recognised-veterinary-practice/.

[16] Baumgaertner H., Mullan S., Main D.C.J. (2016). Assessment of unnecessary suffering in animals by veterinary experts. Vet. Rec..

[17] Fordyce P. (2017). Suffering in non-human animals: Perspectives from animal welfare science and animal welfare law. Glob. J. Anim. Law.

[18] Grimm H., Bergadano A., Musk G.C., Otto K., Taylor P.M. (2018). Drawing the line in clinical treatment of companion animals: Recommendations from an ethics working party. Vet. Rec..

[19] Robertson I.A. (2015). Animals, Welfare and the Law: Fundamental Principles for Critical Assessment.

[20] Schnobel S. (2017). Regulating the veterinary profession: Taking seriously the best interests of the animal. Prof. Neglig..

[21] Birchley G. (2014). Deciding together? Best interests and shared decision-making in paediatric intensive care. Health Care Anal..

[22] Baines P. (2010). Death and best interests: A response to the legal challenge. Clin. Ethics.

[23] S vs. McC; W v W AC 24. https://swarb.co.uk/s-v-mcc-w-v-w-hl-1972/.

[24] Yates and Gard vs. Great Ormond Street Hospital for Children NHS Foundation Trust EWCA Civ 410, 74. https://www.bailii.org/ew/cases/EWCA/Civ/2017/410.html.

[25] Evans and James v Alder Hey Children’s NHS Foundation Trust and Evans Supreme Court. https://www.supremecourt.uk/cases/docs/alfie-evans-reasons-200318.pdf.

[26] *Re T* 1 All ER 906, 916b-c. https://www.bailii.org/ew/cases/EWCA/Civ/1996/1313.html.

[27] Webster J. (1994). Animal Welfare: A cool Eye towards Eden.

[28] Broom D. (2011). A history of animal welfare science. Acta Biotheor..

[29] Yeates J. (2010). Death is a welfare issue. J. Agric. Environ. Ethics.

[30] Jensen K.K. (2017). How should death be taken into account in welfare assessments?. J. Agric. Environ. Ethics.

[31] Rollin B., Regan T., Singer P. (1989). Animal Pain. Animal Rights and Human Obligations.

[32] Regan T. (2004). The Case for Animal Rights.

[33] Christiansen S.B., Kristensen A.T., Lassen J., Sandøe P. (2016). Veterinarians’ Role in Clients’ Decision-Making Regarding Seriously Ill Companion Animal Patients. Acta Vet. Scand..

[34] Linzey A. (2013). Why Animal Suffering Matters: Philosophy, Theology and Practical Ethics.

[35] United Nations Convention on the Rights of the Child, General Comment. https://tbinternet.ohchr.org/_layouts/15/treatybodyexternal/TBSearch.aspx?Lang=en&TreatyID=5&DocTypeID=11.

[36] Spitznagel M.B., Jacobson D.M., Cox M.D., Carlson M.D. (2017). Caregiver burden in owners of a sick companion animal: A cross-sectional observational study. Vet. Rec..

[37] Serpell J. (2019). How happy is your pet? The problem of subjectivity in the assessment of companion animal welfare. Anim. Welf..

[38] Cobb M.L., Lill A., Bennett P.C. (2020). Not all dogs are equal: Perception of canine welfare varies with context. Anim. Welf..

[39] Cairns-Haylor T., Fordyce P. (2017). Mapping discussion of canine obesity between veterinary surgeons and dog owners: A provisional study. Vet. Rec..

[40] Packer R.M.A., Hendrix A., Burn C.C. (2012). Do dog owners perceive the clinical signs related to conformational inherited disorders as ‘normal’ for the breed? A potential constraint to improving canine welfare. Anim. Welf..

[41] *Re Ashya King (a Child)* EWHC 2964 (Fam), 31. https://www.bailii.org/ew/cases/EWHC/Fam/2014/2964.html.

[42] Carlisle-Frank P., Frank J.M. (2006). Owners, Guardians and Owner-Guardians: Differing Relationships with Pets. Anthrozoos.

[43] Charles N., Davies C.A. (2008). My Family and Other Animals: Pets as Kin. Sociol. Res. Online.

[44] Kilkelly U., Sutherland E.E., Macfarlane L.-A.B. (2016). The Best Interests of the Child: A Gateway to Children’s Rights?. Implementing Article 3 of the United Nations Convention on the Rights of the Child: Best Interests, Welfare and Well-Being.

[45] Politi M.C., Street R.L. (2011). The importance of communication in collaborative decision making: Facilitating shared mind and the management of uncertainty. J. Eval. Clin. Pract..

[46] Beauchamp T.L., Childress J.F. (2019). Principles of Biomedical Ethics.

[47] Bell D., Zalta E.N. (2016). Communitarianism. The Stanford Encyclopedia of Philosophy.

[48] Food Ethics Council Ethical Matrix. https://www.foodethicscouncil.org/app/uploads/2019/02/Ethical_Matrix_1.pdf.

[49] Gray C., Fox M., Hobson-West P. (2018). Reconciling autonomy and beneficence in treatment decision-making for companion animal patients. Liverp. Law Rev..

[50] Fordyce P. (2019). A discussion of teaching clinical veterinary ethics to undergraduates: Personal thoughts from the front line. J. Anim. Welf. Sci. Ethics Law Vet. Assoc..

[51] Farm Animal Welfare Council (2009). Appendix IV Ethical principles: How can we decide what is right and what is wrong in the treatment of farm animals?. Farm Animal Welfare in Great Britain: Past, Present and Future.

